# Phenotypic and genotypic analysis of benzimidazole resistance in the ovine parasite *Nematodirus battus*

**DOI:** 10.1186/s13567-014-0116-5

**Published:** 2014-12-09

**Authors:** Alison A Morrison, Sian Mitchell, Rebecca Mearns, Iain Richards, Jacqui B Matthews, David J Bartley

**Affiliations:** Moredun Research Institute, Pentlands Science Park, Bush Loan, Penicuik, EH26 0PZ UK; Animal Health and Veterinary Laboratories Agency, Camarthen, Job’s Well Road, Johnstown, Camarthen, SE31 3EZ UK; Animal Health and Veterinary Laboratories Agency, Penrith, Merrythought, Calthwaite, Penrith, Cumbria, CA11 9RR UK; Care of Westmorland Veterinary Group, Riverside Business Park, Natland Road, Kendal, LA9 7SX UK

## Abstract

Benzimidazole resistance is common amongst many ovine trichostrongylid nematodes species globally. Although anthelmintics have been used for over half a century in some areas of the world for the control of *Nematodirus battus*, resistance has never been detected. Veterinary investigations conducted in 2010 demonstrated reduced efficacy in a flock that had been treated previously with fenbendazole (FBZ), suggesting probable resistance in *N. battus*. Infective larvae (L_3_; designated MNba2) were generated from the original material to conduct a controlled efficacy test (CET). Faecal egg counts showed an average of 37% reduction in the FBZ treated group 7 days post treatment compared to the untreated lambs. Average worm burden results showed no reduction after FBZ treatment compared to the untreated group (3850 and 3850 worms respectively). A molecular assay to assess the frequency of the commonly associated single nucleotide polymorphisms (SNP) in the β-tubulin isotype 1 gene, F200Y and E198A, was developed. Larval genotypes were predominantly homozygous resistant at codon 200 SNP, ranging from 56%-83% and remained stable at 70% for adult worm populations taken from treated and control lambs in the CET. Only susceptible genotypes were found at codon 198. The allele frequency for F200Y ranged between 80-83% in adult worms taken from the CET from treated and control lambs. The results confirmed initial findings and demonstrated the first report of FBZ resistance in *N. battus* whilst providing evidence that the P200 point mutation in the β-tubulin isotype 1 gene is a potential mechanism of resistance in the species.

## Introduction

There are several species of *Nematodirus* that cause disease and production losses in young lambs, although most of the losses in the UK are caused by *Nematodirus battus* [1-3]. Nematodirosis is a disease that is normally seen in young lambs, the signs of infection are often acute and, if left unchecked, can result in a high (typically 5-20%) mortality rate [3]. Acute disease is the consequence of very heavy larval challenge and the effects of the developing larvae. As a result, lambs can present with severe scouring and other clinical signs before there are well established levels of adult worms and, hence, eggs appearing in faeces*.* Currently five classes of broad spectrum anthelmintics are available for use in sheep in the UK. All classes have licensed efficacy against *N. battus*, however, activity against certain life stages (particularly immatures) can vary by formulation and mode of application [4], particularly within the macrocyclic lactone class. Many farmers in the UK opt to use benzimidazoles due to its wide safety margin and good efficacy [5]. Reports of resistance to this class of anthelmintic are commonplace in ovine trichostrongylid nematode species such as *Teladorsagia circumcincta* throughout sheep flocks in the UK [6-9] and globally [10-14]; however it has never previously been reported in *N. battus*. The only reports of benzimidazole resistance involving *Nematodirus* spp. (*Nematodirus spathiger*) are cases of oxfendazole resistance in New Zealand, Australia and Tasmania [15-17] and one case of thiabendazole resistance in a mixed species isolate of *N. spathiger* and *Nematodirus filicollis* in Australia [18].

Several genetic mechanisms have been associated with BZ resistance in parasitic nematodes of sheep; for example, loss of isotype 2 of the β-tubulin gene and single nucleotide polymorphism (SNP) within isotype 1 of the β-tubulin gene [19,20]. SNPs in the β-tubulin gene are responsible for an amino acid transversion at each of the sites; phenylalanine to tyrosine at codon 200 (TTC → TAC, F200Y; [21,22], phenylalanine to tyrosine at 167 (TTT → TAT, F167Y; [23,24] and glutamic acid to alanine at 198 (GAA → GCA, E198A; [25]). Not all of these mutations are found in all ovine parasitic nematode species that are resistant and the presence of one SNP is not usually accompanied by a second [26]. At present, no information relating to the mechanisms involved in benzimidazole resistance in *N. battus* is available.

In 2010, a clinical case of nematodirosis was submitted to Animal Health Veterinary Laboratory Agency (AHVLA) Penrith where a farmer was concerned about a group of 60 lambs showing signs of disease (diarrhoea and ill thrift); three lambs had died from the group, with a fourth being submitted for post mortem examination. Nematodirosis due to *N. battus* was diagnosed as the cause of death due to the high numbers of *N. battus* eggs detected in the faeces (1850 eggs per gram) and a small number (300) of adults present in the small intestinal contents. No other gastro-intestinal nematodes were identified in the abomasal or small intestinal contents. Treatment of the surviving lambs with FBZ resulted in little clinical improvement; however the farmer only treated the lambs to the average weight of the group. After seeking further advice, a faecal egg count reduction test (FECRT) was conducted by the attending veterinarian using FBZ at the manufacturer’s recommended dose rate (MRDR) and faecal samples were submitted to the AHVLA at the time of treatment (designated hereafter as MNba2^VCF^) and 10 days later [27].

Here, the authors report the findings from a controlled efficacy test (CET) performed at Moredun Research Institute using the field isolate of *N. battus* derived from the MNba2^VCF^ generated material. Secondly, they describe a pyrosequencing assay developed to identify polymorphisms at codons 198 and 200 of the β-tubulin isotype 1 gene to investigate the potential mechanisms involved in BZ resistance in *N. battus*.

## Materials and methods

### Parasite isolate

Faecal material was collected from lambs following an on-farm FBZ FECRT which indicated the presence of benzimidazole resistance [27]. *N. battus* eggs were cultured and extracted from faeces using a modification of methodologies described in the Manual of Veterinary Parasitology Techniques reference book [28]. In brief, eggs were extracted from faeces by differential sieving and salt flotation. Recovered eggs were stored in tap water in 75 cm^2^ vented culture flasks (Corning B.V. Life Sciences, Amsterdam, The Netherlands) at around 20 °C for over 7 weeks to allow full development of larvae in the eggs. To assist hatching, eggs were sandwiched between two glass plates and gently crushed by applying downward pressure until a faint cracking sound was heard. Successful hatching was assessed by viewing the eggs down a stereo microscope (×20) before Baermannising the suspension. The resultant third stage larvae (L_3_ n = 4300) were subsequently passaged through a parasite-naïve lamb, which was administered with FBZ at day 43 post-infection (pi) at MRDR. Faecal material was collected before and after treatment and processed as above to generate sufficient L_3_ for the controlled efficacy trial. Eggs recovered post-treatment were artificially hatched and the L_3_ used in the controlled efficacy trial.

### Experimental design

Ten helminth-free, female, six month-old Texel X Greyface lambs were each artificially infected *per os* with 6000 L_3_ (day 0 pi). On day 24 pi, faecal egg counts (FEC) were conducted using a modification of the salt flotation technique described by [29] and [30] with a sensitivity of up to one egg per gram. All lambs were weighed. The lambs were then allocated into one of two groups ensuring they were balanced as closely as possible for both FEC and weight. On day 25 pi, one group was orally administered fenbendazole (Panacur 2.5%, Intervet, Milton Keynes, UK; 5 mg/kg body weight (BW)), whilst the second group remained untreated. These groups were designated MNba2^FBZ^ and MNba2^CON^, respectively. All anthelmintic treatment doses were calculated according to the respective manufacturer’s instructions, with doses rounded up to the nearest 0.5 mL (dosage range 5.0 - 5.3 mg/kg BW). Faecal samples were taken *per rectum* daily from each lamb from day 14 pi until day 32 pi and FECs conducted. All lambs were slaughtered on day 32 pi and the small intestines removed for saline digest to estimate total worm burdens. Methods used for necropsy and worm recovery were as described previously [31]. Total burdens of each animal were estimated from a 2% subsample (100 mL) of the small intestinal wash and digest. Worms were separated into adult males, adult females and juvenile stages using the criteria described in the Manual of Veterinary Parasitology Techniques reference book [28].

All experimental procedures conducted at Moredun Research Institute were assessed and approved by the Institute’s Experiments and Ethics Committee and were conducted under the legislation of a UK Home Office License (reference PPL 60/03899) in accordance with the Animals (Scientific Procedures) Act of 1986.

### Statistical analysis

Nematode burdens and FECs were square-root transformed to normalize for variance. Burdens were compared using one way ANOVA (Minitab version 13, Coventry, UK), followed by Fisher’s pairwise comparisons when found to be significant (*p* < 0.05). Statistical significance was accepted as *p* < 0.05. Fenbendazole efficacy was calculated based on group mean FECs using one of a range of standard formulae, (1 − [T2/C2]) × 100 using arithmetic means [32], (1 − [T2/T1] [C1/C2]) × 100 using geometric means [33], (1 − [T2/T1] [C1/C2]) × 100 using arithmetic means [34], (1 − [T2/T1]) × 100 using arithmetic means [35], where C1 and C2 are the FEC of untreated control animals pre- and post treatment respectively and T1 and T2 are the FEC of animals pre- and post treatment respectively. Bootstrap analysis of the data using the “BootStreat” program was also conducted, with a re-sampling number of 2000 to calculate mean treatment efficacies and upper and lower 95% confidence limits [36]. Efficacy based on total worm burdens was calculated using the WAAVP guidelines formula [32].

### Molecular analysis

Due to lack of published sequences available for *N. battus* β-tubulin gene, partial β-tubulin isotype 1 sequences were amplified from cDNA generated from total RNA that had been extracted using TRIzol reagent (Life Technologies, California, USA) from 100, 000 L_3_ from a known FBZ-sensitive isolate (designated MNba1) and MNba2^VCF^ isolate L_3_ obtained after FBZ treatment along with a FBZ susceptible isolate of *Haemonchus contortus* (MHco3; [37]), as a control. Twenty microlitres of cDNA were generated using Invitrogen superscript III reverse transcriptase kit (Invitrogen, California, USA), where 4 μL of RNA extract was used per sample. Generic primers (Table 1: Gen Beta-tub For1 & Gen Beta-tub Rev1) were designed to amplify an area of the isotype 1 β-tubulin gene that covered the three most common SNP’s of interest using published sequences from other trichostrongylid nematode species; *Trichostrongylus colubriformis* (Accession number: L23506), *Teladorsagia circumcincta* (Accession number - Z69258), *H. contortus* (Accession number - M76491/EF198865) and *Cooperia oncophora* (Accession number - AY259994). PCR products were amplified using Novataq™ Hot start master mix (Novagen, Madison, USA) in a 50 μL reaction, with primers and MgCl_2_ at a final concentration of 0.3 μM and 3 mM, respectively, with the following cycle conditions: 94 °C for 10 min, (94 °C for 30 s, 52 °C for 30 s, 72 °C for 30 s) for 35 cycles and 72 °C for 10 min. Beta-tubulin isotype 1 sequences spanning the SNPs of interest were also generated from genomic DNA (gDNA) isolated from 10 individual adult *N. battus* worms (5 MNba1 and 5 MNba2^CON^). One microlitre of gDNA was added to a 25 μL PCR reaction using HcPy2 PCR For and HcPy2PCR Rev primers [38], see Table 1) at 0.3 μM and 3.0 mM MgCl_2_ final concentrations in NovaTaq™ Hotstart master mix. PCR amplicons of the appropriate size were cut from a 1.5% agarose gel (Gel Extraction Kit, Qiagen, Hilden, Germany) and the gDNA ligated into the pGEM-T vector (Promega, Madison, USA) following the manufacturer’s protocol. Transformations were carried out following the standard protocol for JM109 *Escherichia coli* Competent Cells (Promega) and two (for gDNA sequences) and six (for cDNA sequences) transformed colonies were picked and grown up in 10 mL LB broth overnight before DNA extraction was carried out using a Wizard SV mini prep kit (Promega). DNA concentrations for gDNA and cDNA were assessed using a Nanodrop spectrophotometer (Nanodrop Technologies Inc., Delaware, USA) and adjusted if necessary using DNA/RNA-free water according to Eurofins MWG requirements for sequencing. DNAStar (Madison, USA), Lasergene 9 EditSeq, BLAST and ClustalW were used to analyse and align sequences. Consensus *N. battus* sequence was obtained using the gDNA sequences and used to design primers that spanned codons 200 and 198 for a pyrosequencing assay using “Pyrosequencing assay design” software, Version 1.0 (Table 1).

**Table 1:** Primers used in this study (*invitrogen oligo perfect designer used; # pyrosequencing assay design software version 1.0 used; ^**$**^ **primer sequences obtained from** [38]

**Oligo**	**5′** **3′**
Gen Beta-tub For1*	ATGCGTGARATCGTYCAY
Gen Beta-tub Rev1*	CGAGGGAARGGKACCAT
HcPy2 PCR For^$^	GACGCATTCACTTGGAGGAG
HcPy2PCR Rev^$^	Biotin-CATAGGTTGGATTTGTGAGTT
Nb B-t200 Fbio^#^	Biotin- AGGTAGGTGTGGCCTATCAAAAT
Nb B-t200 Rev^#^	ATGTTCGGAAACAGATGTCGTAC
Nb B-t200 Seq^#^	TTCGTTGTCAATGCAG

#### Pyrosequencing

To provide gDNA for pyrosequencing, 20 ethanol-fixed adult *N. battus* were used from three populations; MNba1, MNba2^CON^ and MNba2^FBZ^. Genomic DNA was extracted using DNeasy Kit (Qiagen) as per kit instructions. For PCR, the adult gDNA extracts were diluted 1:5 in DNA/RNA-free water (Sigma, Dorset, UK). For provision of gDNA from four populations of L_3_ (MNba1, MNba2^VCF^, MNba2^CON^ and MNba2^FBZ^), ethanol fixed L_3_ were bathed in PBS for 15–30 min prior to transferring individuals into 30 μL of lysis buffer (50 mM KCl, 10 mM Tris (pH 8.3), 2.5 mM MgCl_2_, 0.45% (v/v) Nonidet P-40, 0.45% (v/v) Tween 20, 0.01% (w/v) gelatin and 0.1 mg/mL Proteinase K) in a 96-well plate (Axygen, California, USA); frozen for 30 min at −20 °C, prior to incubating at 56 °C overnight. Ethanol precipitation was carried out to clean up the gDNA and re-supsended in 25 μL of DNA/RNA-free water (as above). A minimum of 80 L_3_ were lysed for each study population. For pyrosequencing, 4 μL of gDNA from both adult and L_3_ were amplified in a mix containing 0.185 μM Nb B-t200 Fbio, 0.2 μM Nb B-t200 Rev, 4.5 mM MgCl_2_, 25 μL 2 × buffer and made up to 50 μL using DNA/RNA-free water (NovaTaq™ Hot start master mix, Novagen). No template controls were included on each plate. Amplification was performed, following a 15 min 95 °C polymerase activation step, for 45 cycles at 94 °C for 30 s, 58 °C for 30 s and 72 °C for 30 s, followed by a final extension step at 72 °C for 10 min. The pyrosequencing P200/198 assay was conducted according to the manufacturer’s (Qiagen) protocols using a PyroMark ID instrument. In brief, following PCR amplification, 40 μL of the reaction was transferred into a 96-well plate and stored at 4 °C until the remaining PCR reaction (10 μL) was assessed by gel electrophoresis on 2% agarose gels stained with gel red (Biotium, California, USA). The plate was run on the Pyromark ID instrument if clear PCR amplification was observed.

#### Genotyping/allele frequency

Genotypes (TTC/TTC, TTC/TAC and TAC/TAC) and allele frequencies for resistant (R) and susceptible alleles (S) from each population are expressed as percentages [39].

Chi–square tests and Phi coefficient association of P200 genotypes were conducted using Minitab version 15.

## Results

### Faecal egg count reduction test analysis

**Figure 1:**
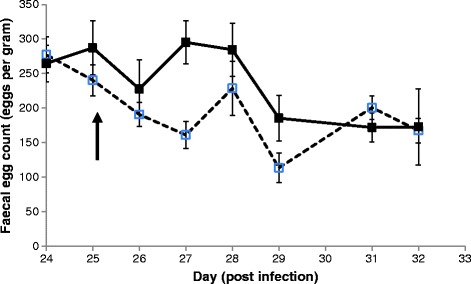
**Faecal egg count profile of lambs infected with 6,000** ***Nematodirus battus*** **infective larvae.** Arithmetic mean faecal egg counts (± standard error of the mean) of two groups of lambs infected with 6000 *Nematodirus battus* infective larvae. On day 25 post infection (↑) one group was orally administered fenbendazole (□; Panacur, Intervet; 5 mg/kg BW), whilst the second group remained untreated (■).

All individual lambs were excreting *N. battus* eggs by day 14 pi. The FEC counts continued to rise in most animals until day 24 pi (Figure 1). Arithmetic mean FECs at day 24 pi were 265 (range 198 – 338 EPG) and 277 (range 216 – 360 EPG) EPG for the MNba2^CON^ and MNba2^FBZ^ groups, respectively. At post mortem the mean FEC of both groups remained similar, with 167 and 172 EPG for the FBZ treated and control groups, respectively, Table 2. No significant difference was observed between the groups.

**Table 2:** **Arithmetic mean (± S.E.M) [range] faecal egg counts of untreated control and fenbendazole treated lambs on day of treatment and seven days post treatment**

**Treatment**	**Faecal egg count (eggs per gram)**		Percentage efficacy (95% CI)*			
	**Day 0**	**Day 7**	**1**	**2**	**3**	**4**
Untreated	287 (±39) [203–410]	173 (±55) [32–369]	-	-	-	-
Fenbendazole	240 (±22) [162–293]	167 (±18) [117–221]	3 (0, 30)	30 (8, 47)	0 (0, 11)	30 (28, 31)

*Percentage efficacy with upper and lower confidence intervals calculated using four different methodologies 1 - [32]; 2 - [33]; 3 - [34]; 4 - [35].

### CET

**Figure 2:**
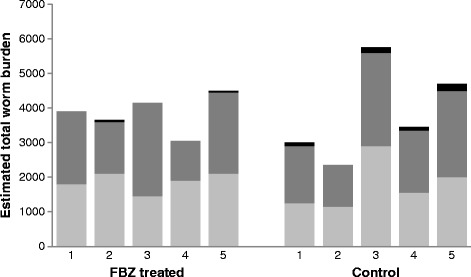
**Individual estimated total worm burden of the two groups of lambs infected with 6000** ***Nematodirus battus*** **infective larvae.** The stacked bar chart shows the estimated total worm burden (reported as males (light grey), females (dark grey) and juveniles (black)) for each lamb in the fenbendazole (FBZ) treated (MNba2^FBZ^; 5 mg/kg FBZ body weight) and control (MNba2^CON^) groups. The control group (MNba2^CON^) estimated total number of worms ranged between 2350 and 5750, with an arithmetic mean of 3850 worms. The FBZ treated group (MNba2^FBZ^) estimated total number of worms ranged between 3050 – 4500 worms, with an arithmetic mean of 3850 worms. This showed a 0% efficacy with FBZ treatment.

Arithmetic mean percentage establishment of *N. battus* in control lambs (MNba2^CON^) was 64% (arithmetic mean = 3850 worms), with the estimated total number of worms ranged between 2350 and 5750 (Figure 2) within the group. Arithmetic mean worm burden of the FBZ treated (MNba2^FBZ^) group was 3850 worms (range, 3050 – 4500 worms) and hence the treatment efficacy was 0%.

### Molecular analysis

#### Sequencing results

A 785 bp fragment was amplified from the cDNA from the resistant (MNba2^VCF^) and susceptible (MNba1) isolates of *N. battus*. Sequences from MNba1 were subjected to a Blast search, with sequence homologies of 94% with *T. circumcincta* (Z69258) and 85% with *C. oncophora* (AY259994) isotype 1 sequences with hits also showing homology with, *H. contortus*, *O. ostertagi* and *T. colubriformis* isotype 1 β-tubulin sequences. Consensus sequence derived from the MNba2^VCF^ isolate showed 87% homology to *H. contortus* and *C. oncophora* (AY259994) isotype 1 β-tubulin mRNA sequences. A smaller fragment of approximately 340 bp was amplified from genomic DNA. The consensus sequence from MNba1 gDNA blast search showed 86% and 85% homology with *H. contortus* (FJ981633) and *T. circumcincta* (Z69258) isotype 1 β-tubulin sequences respectively. Similar results were produced with MNba2^FBZ^ consensus sequence.

#### Genotyping

Cross reactivity of the isotype 1 β-tubulin pyrosequencing assay was examined against other species, *N. filicollis*, *T. circumcincta*, *H. contortus* and *T. colubriformis*. DNA from these species did not provide amplification using the Nb B-t 200 forward and reverse primers. The mean percentage genotypes (homozygous TTC/TTC, heterozygous TTC/TAC and homozygous TAC/TAC) for L_3_ and adult worms from the MNba1 and MNba2 populations are shown in Tables 3 and 4, respectively. For all samples tested, the MNba2 isolate was predominantly homozygous resistant genotype (TAC/TAC) at codon 200: for example, 56% of L_3_ (MNba2^VCF^) were homozygous resistant in the population obtained from the on-farm FECRT and 83% of L_3_ (MNba2^CON^) from the CET control population were homozygous resistant. In the case of the adult worms obtained from the CET study, 70% of all worms tested in the (MNba2^CON^ and MNba2^FBZ^) populations were identified as homozygous resistant at codon 200.

**Table 3:** **Beta-tubulin codon 200 genotyping results of the infective larvae of two** ***Nematodirus battus*** **isolates (MNba1 FBZ-sensitive and MNba2, FBZ-resistant phenotypes)**

**Population**	**Number of larvae examined**	**Relative proportion of codon 200 genotype (%)**			**Allele frequency (%)**	
		**TTC/TTC**	**TTC/TAC**	**TAC/TAC**	**S**	**R**
MNba1	95	100	0	0	100	0
MNba2^VCF^	98	10	34	56	27	85
MNba2^CON^	81	2	15	83	10	90
MNba2^FBZ^	103	3	20	77	13	87

**Table 4:** **Beta-tubulin codon 200 genotyping results from** ***Nematodirus battus*** **adult worms of two** ***Nematodirus battus*** **isolates (MNba1 FBZ-sensitive and MNba2, FBZ-resistant phenotypes)**

**Population**	**Number of worms**	**Relative proportion of codon 200 genotype (%)**			**Allele frequency (%)**	
		**TTC/TTC**	**TTC/TAC**	**TAC/TAC**	**S**	**R**
MNba1	20	100	0	0	100	0
MNba2^CON^	20	5	25	70	18	83
MNba2^FBZ^	20	10	20	70	20	80

Both L_3_ and adult worms derived from the MNba2^FBZ^ samples showed a slightly higher number of homozygous susceptible genotypes (TTC/TTC) compared to the MNba2^CON^ population; the Chi-square test showed that there was no significant difference (adults - *X*^2^ = 0.44, df = 2, *p* = 0.801 and L_3_ – *X*^*2*^ = 1.025, df = 2, *P* = 0.599) between the genotypes of these populations. The test showed a difference between the P200 genotypes identified, when worms derived from MNba2^VCF^ and MNba2^CON^ (*X*^2^ = 14.833, df = 2, *p*-value = 0.001) were compared. No individuals from isolate MNba1 were homozygous (TAC/TAC) or heterozygous (TTC/TAC) resistant at codon 200. Only homozygous “susceptible” genotypes (GAA/GAA) were identified at codon198 for both adults and larval populations from all the *N.battus* isolates examined here.

The R allele frequency for 200Y ranged from 85% - 90% within the MNba2 L_3_ populations (MNba2^VCF^, MNba2^CON^ and MNba2^FBZ^; see Table 3). The R allele frequency was 83% and 80% for MNba2^CON^ and MNba2^FBZ^ adults, respectively (see Table 4).

## Discussion

The results here provide confirmation, based on residual worm burden and faecal egg count analysis post benzimidazole treatment, of the preliminary results of the first reported case of BZ resistance in *N. battus* globally [27]. The zero percentage efficacy noted here, poses the question as to whether any members of the benzimidazole class of anthelmintics would be effective against this isolate. In this context, side resistance within the BZ class has been shown to occur in *T. colubriformis*, *H. contortus* [40,41] and undifferentiated *Nematodirus* species [18] and may be expected to occur with *N. battus.* Currently, the most practical method of determining anthelmintic sensitivity is the FECRT. There are a number of drawbacks to this test; it is time-consuming, requiring 10–14 days to complete [42], is relatively insensitive at low levels of resistance [43] and is potentially prone to misdiagnosis if the window of opportunity prior to re-infection is missed. With *Nematodirus,* in particular, the correlation between FEC and worm burden is particularly poor in treated [44] and untreated lambs [45,46]. *Nematodirus battus* historically was characterised as being fully developed by 14 days pi, but that maximum egg production did not occur until slightly later [47]. The results based on the isolate here suggest that significant FEC may be detectable in faeces on or before 14 days pi, even at moderate infection rates. The current findings are contrary to those found in New Zealand where in some cases of BZ resistance in *N. spathiger*, adult worms were recovered in animals that had zero FEC after BZ administration [48]. If the disparity between FEC and worm burden can occur with *N. battus* then it may explain the lack of detection of resistance in this parasite in the past and would have a bearing on detection of resistance in the field.

To implement sustainable control strategies it is essential to identify the presence of resistance at an early stage of development. In vitro detection of benzimidazole resistance in *Nematodirus* species using traditional methodologies such as the egg hatch test is complicated by the parasites development and hatching behavior as well as the egg shell morphology [16]. Therefore to better understand the acquisition and development of FBZ resistance in *N. battus* the authors here strove to identify whether any of the “commonly associated” point mutations of the β-tubulin isotype 1 gene were present in the FBZ resistant population. The results from MNba2 only yielded the F200Y SNP and found that there was no changes observed at E198A. The relationship between F200Y mutations in *N. battus* and actual anthelmintic efficacy in the field requires further examination, but numerous studies have shown that in other trichostrongylid species there is a high degree of correlation between phenotypic BZ characterisation and resistance allele frequencies of various life stages [49-52]. Researchers have found that *H. contortus* individuals which are homozygous resistant at codon 198 have a higher level of phenotypic resistance than those with SNP mutations at codon 200 [51]. Further work is required in *N. battus* to investigate other SNPs in the isotype 1 gene and also to characterize isotype 2 of the β-tubulin gene as it is possible that this is present in the populations here as indicated by amplification of some sequences with high identity to this isotype (data not shown).

The comparison of the genotyping results obtained from the MNba2 isolate over the course of the study, starting from the initial field isolate (MNba2^VCF^) to the CET populations (MNba2^CON^ and MNba2^FBZ^), showed that the two treatments with FBZ at the MRDR (one in the donor animal that produced the L_3_ for the CET and one as part of the CET) only marginally led to a difference in the F200Y allele frequency (85, 90 and 87% respectively), which would indicate that this population is stable and highly selected. The observation of a small number of RS and SS genotypes in adult survivors following FBZ treatment, suggests either that the worms are phenotypically sensitive but were able to “hide” from the lethal effects of treatment and then recover/resume activities as the local anthelmintic concentrations fall over time, or that there are other potential mechanisms of resistance involved in the BZ resistant phenotype. These findings are in agreement with other characterisation studies where analysis of the genotypes of first stage *T.circumcincta* larvae survivors from an in vitro egg hatch test, at concentrations up to 2 μg/mL thiabendazole, still had around 5% homozygous susceptible and 15% heterozygous genotypes [53].

As to why this isolate of *N. battus* developed FBZ resistance when previously the species has appeared to be susceptible to treatment is unclear and may be the result of a number of factors. It is possible that detection on this farm was aided by greater awareness of anthelmintic resistance and good co-operation between the farmer and his veterinarian. Benzimidazoles had been used on the farm to control *N. battus* since 2007 until resistance was diagnosed in 2010. Inadvertent under-dosing could have occurred as lamb weight was estimated and they did not normally calibrate their dosing equipment. Reduced bioavailablity due to rapid gut flow may also have contributed to under-dosing [54,55] either from the clinical effects of *N. battus* or other concurrent causes of diarrhoea, such as coccidiosis. One of the major pathogenic effects of *N. battus* is attributable to disruption of the intestinal mucosa and the villous atrophy associated with larval stage development. The result of this atrophy is a reduced capability for fluid exchange, acute diarrhoea and increased gut flow [56]. Under dosing has been shown to allow heterozygote resistant individuals to survive treatment and contribute genes for resistance to the subsequent populations [57,58].

In some years benzimidazole treatments had been repeated at approximately monthly intervals in the young lambs on this farm dependent on the farmer and veterinary surgeons perception of risk of disease. Prophylactic treatments three weeks apart have been recommended in high risk areas to coincide with the predicted timing of peak hatch [56]. These high treatment frequencies place a considerable selection pressure on the population and could have, over time, resulted in the development of a resistant population.

As common on many sheep farms successive lamb crops grazed the same permanent pastures year on year. This in combination with frequent treatment administrations may have resulted in the slow increase in gene frequency for FBZ resistance within the population. There was no history of dose and movement of lambs onto new pastures. Previous studies have shown that rapid selection of BZ resistance in nematode species has occurred following dose and move treatment strategies [59-61], if lambs are grazed on pastures that had been previously been “seeded” with eggs of survivors of FBZ treatment, the possibility exist that resistance could be selected and propagated [60,62].

Changes in the parasite population brought about by changes in environmental/climatic conditions leading to changes in human behaviour, land use and/or animal husbandry [63-65] could be involved. Changes in climate have been shown to lead to conditions favourable for longer grazing periods [66]. The potential for longer parasite seasons will inevitably result in subsequent changes in treatment patterns associated with controlling infections leading to possible parasite adaptation.

However, no significant risk factor(s) can be identified on this farm with respect to anthelmintic resistance development in *N. battus* that would not be present on a large number of other sheep farms in the UK. It may be that within *N. battus*, the polymorphism associated with FBZ resistance conferred a fitness cost and until the introgression of resistance gene(s) within a population was sufficiently stable, the population was unable to thrive and propagate itself leading to a sporadic appearance on the farm. Previous works conducted on other nematode species, namely *H. contortus* and *T. colubriformis*, have observed poorer development and survival of eggs in anthelminitic resistant populations at a variety of temperatures [67,68].

An understanding of whether selection for BZ resistance within this population has occurred as a result of pre-adaptation, spontaneous mutation or gene flow will provide a better opportunity to develop effective sustainable strategies for control and now requires further investigation. This study highlights that for sustainable control of *N. battus,* farmers need to consider monitoring treatment efficacies, to minimize treatment frequency where possible and avoid indiscriminate use of anthelmintic compounds.
